# Prediction of a positive surgical margin and biochemical recurrence after robot-assisted radical prostatectomy

**DOI:** 10.1038/s41598-021-93860-y

**Published:** 2021-07-12

**Authors:** Ching-Wei Yang, Hsiao-Hsien Wang, Mohamed Fayez Hassouna, Manish Chand, William J. S. Huang, Hsiao-Jen Chung

**Affiliations:** 1 Department of Urology, Cheng-Hsin General Hospital, No. 45, Cheng-Hsin St., Beitou Dist., Taipei, 112 Taiwan; 2 Department of Urology, College of Medicine and Shu-Tien Urological Research Center, National Yang Ming Chiao Tung University, No.155, Sec.2, Linong Street, Taipei, 112 Taiwan; 3 Wellcome EPSRC Centre for Interventional and Surgical Sciences (WEISS), University College London, 43-45 Foley Street, London, W1W 7JN UK; 4 Department of Colorectal Surgery, University College London Hospital, 250 Euston Road, London, NW1 2BU UK; 5 Department of Urology, Taipei Veterans General Hospital, 201, Sec 2, Shih-Pai Rd., Beitou Dist., Taipei, 112 Taiwan; 6 Division of Surgical and Interventional Sciences, Charles Bell House, 43-45 Foley St, Fitzrovia, London, W1W 7TY UK

**Keywords:** Prostate cancer, Surgical oncology, Disease-free survival, Urology

## Abstract

The positive surgical margin (PSM) and biochemical recurrence (BCR) are two main factors associated with poor oncotherapeutic outcomes after prostatectomy. This is an Asian population study based on a single-surgeon experience to deeply investigate the predictors for PSM and BCR. We retrospectively included 419 robot-assisted radical prostatectomy cases. The number of PSM cases was 126 (30.1%), stratified as 22 (12.2%) in stage T2 and 103 (43.6%) in stage T3. Preoperative prostate-specific antigen (PSA) > 10 ng/mL (*p* = 0.047; odds ratio [OR] 1.712), intraoperative blood loss > 200 mL (*p* = 0.006; OR 4.01), and postoperative pT3 stage (*p* < 0.001; OR 6.901) were three independent predictors for PSM while PSA > 10 ng/mL (*p* < 0.015; hazard ratio [HR] 1.8), pT3 stage (*p* = 0.012; HR 2.264), International Society of Urological Pathology (ISUP) grade > 3 (*p* = 0*.*02; HR 1.964), and PSM (*p* = 0.027; HR 1.725) were four significant predictors for BCR in multivariable analysis. PSMs occurred mostly in the posterolateral regions (73.8%) which were associated with nerve-sparing procedures (*p* = 0.012) while apical PSMs were correlated intraoperative bleeding (*p* < 0.001). A high ratio of pT3 stage after RARP in our Asian population-based might surpass the influence of PSM on BCR. PSM was less significant than PSA and ISUP grade for predicting PSA recurrence in pT3 disease. Among PSM cases, unifocal and multifocal positive margins had a similar ratio of the BCR rate (*p* = 0.172) but ISUP grade > 3 (*p* = 0.002; HR 2.689) was a significant BCR predictor. These results indicate that PSA and pathological status are key factors influencing PSM and BCR.

## Introduction

Up to 2019, an estimated 4986 robotic systems have been installed in medical centers worldwide, of which 561 are located in Asian countries^1^. Robot-assisted radical prostatectomy (RARP) has become a standard approach for localized prostate cancer (PCa) treatment^2^. This method yields comparable oncological outcomes as previous open and laparoscopic methods, primarily with respect to positive surgical margin (PSM) and biochemical recurrence (BCR), along with enhancements in functional outcomes, including urinary continence and recovery of erectile potency^3–5^.

PSM detected in radical prostatectomy (RP) specimens is considered a poor oncological outcome^6^; however, its long-term effect on mortality remains uncertain^7^. Previous studies have reported several predictors for PSM, including prostate-specific antigen (PSA) concentration, prostate weight^8^, obesity^9^, the histopathological findings from biopsy and RP specimens^10^, surgeon experience^11^, pathologist interpretation^12^, surgical approach^13^, and surgical method^14–17^, may potentially influence postoperative PSM. However, data from Asian countries regarding the prediction of PSM and BCR are still lacking owing to differences in PCa phenotypes between individuals in Asian and Western countries^18^. It remains difficult for surgeons to determine the risk of PSM before surgery and the effect of PSM on the BCR rate after surgery. In this study, we used case-cohort data from an Asian medical center to further the current understanding of PSM and BCR after RARP.

## Material and methods

### Patients and study design

A medical record of 419 patients who underwent RARP from December 2010 to January 2018 at Taipei Veterans General Hospital, Taiwan, was analyzed retrospectively in this study. The research protocol was approved, and the need for informed consent was agreed to be waived by the Institutional Review Board and Human Research Protection Center of Taipei Veterans General Hospital (No.: 2020-05-001BC). The patient records were anonymized and de-identified before analysis. All study procedures involving data collection and management were in accordance with relevant guidelines and regulations. The indication for prostatectomy was clinically localized prostate cancer revealed through prostate biopsy. The clinical stage was mainly decided by magnetic resonance imaging (MRI) scan. Most patients had undergone MRI at either our center or other hospitals. The MRI scan was performed at least 6 weeks after prostate biopsies to decrease the interference of interpretation. One radiologist reported the MRI stage and the other urology radiologist reconfirmed the results at the urology-radiology combined conference. The MRI scoring system was based on the Prostate Imaging-Reporting and Data System (PI‐RADS) version 1 since 2012 and following the new PI‐RADS version 2 in 2015. We preoperatively collected the following clinical data: age, body mass index (BMI), PSA, International Society of Urological Pathology (ISUP) grade, and cT stage. The following intraoperative parameters were noted: console time, estimated blood loss, previous transurethral resection of the prostate (TURP), previous abdominal surgery, prominent prostate median lobe (observed by the surgeon), number of surgical cases, and the use of nerve-sparing (NS) methods (unilateral or bilateral for the cases without obvious extracapsular extension defined by MRI findings). The following postoperative parameters were recorded: margin status, ISUP at RP specimens, pT stage, prostate weight, Clavien complication grades, and the point of PSA recurrence within five years (defined as PSA level ≥ 0.2 ng/mL in two separate measurements). The locations of PSM were classified as apical, bladder neck, posterolateral, unifocal, and multifocal areas to evaluate the predictive risks. At least two qualified pathologists interpreted the final RP pathological reports.

Exclusion criteria: patients with a history of neoadjuvant hormone therapy or any focal treatment, including radiotherapy, cryotherapy, high-intensity focused ultrasound therapy, and salvage prostatectomy, were excluded. Furthermore, patients lost to follow-up within two months of RARP, and those who received adjuvant therapy owing to adverse pathological outcomes before PSA relapse were excluded.

Preoperative PSA levels were determined immediately before the prostate biopsy, and postoperative PSA levels were determined within 1-month post-biopsy and then at 3-month intervals until PSA recurrence was confirmed. Time zero marked the date of RARP, and patients without BCR did not present PSA recurrence on the most recent follow-up evaluation before the end of 2019 (the observation period from 2 to 115 months). An increase in serum PSA levels was identified twice, and other factors potentially elevating PSA levels were excluded.

### RARP

All RARPs were performed by a single urologist (H.J.C) who had > 15 years’ experience in laparoscopic urological surgery. The surgeon started RARPs in 2010 and performed around 50 cases per year. The surgical technique was aiming to preserve peri-prostatic adjacent structures (endopelvic fascia, dorsal vein complex, bladder neck, and neurovascular bundle, etc.). The surgical procedure only had a little adjustment along the study period. Most of the patients recovered zero pad at the first year of follow-up. For RARP, the transperitoneal approach was adopted, employing the Da Vinci Surgical System Si or Xi with six ports. The prostatic anterior fat pad was removed to visualize the prostatic boundaries. The bladder neck was opened and separated from the prostate. By dividing the vesicoprostatic muscle, the vas deferens and seminal vesicles were exposed. Bilateral vas deferens were transected and then pulled anteriorly to facilitate the dissection of seminal vesicles. The Denonvilliers’ fascia was identified, and the posterior plane was carefully dissected from the base to the prostate apex to preserve neurovascular bundles. The prostatic apex was dissected, thus maximally preserving the urethral stump. The apex was laterally dissected from the anteromedial components of the levator ani. The urethra was posteriorly incised, and the prostate was removed. Pelvic lymph node dissection was done with limited dissection between the external iliac vein and above the obturator nerve. The vesicourethral anastomosis was conducted using a continuous suture. Finally, the incised detrusor apron was reapproximated.

### Statistical analysis

The SPSS Statistics 20 was used for statistical analysis. Pearson’s chi-square and independent samples *t-*test were used for assessing categorical and continuous data, respectively. The significantly different predictors of PSM in univariable analysis (*p* < 0.05)were further compared through multivariable logistic regression analysis. The Kaplan–Meier method was used to estimate the 5-year BCR-free survival rate, and the log-rank test was performed to compare the correlations between each factor and BCR-free survival. The significantly different predictors of BCR in univariate analysis (*p* < 0.05) were pooled together and analyzed by the multivariable Cox proportional hazards regression model. Finally, four positive predictors were compared and analyzed multivariable by stratified pathological stage and surgical margin status.

## Results

### General characteristics

In total, 419 patients who underwent RARP were assessed herein (Table 1). Overall, 181 (43.4%) patients were pT2 stage and 236 (56.6%) were pT3 stage. The upgrading rate of the ISUP grade between biopsy and final RP was significantly higher at stage T3 (49.6%) than at stage T2 (34.8%; *p* = 0*.*003).

**Table 1:** Baseline characteristics of 419 patients treated with robotic prostatectomy.

Characteristics	Median (IQR)
Age, year, median (IQR)	66 (62–60)
BMI, kg/m^2^, median (IQR)	25.2 (23.3–27.2)
Preoperative PSA level, ng/ml, median (IQR)	8.7 (6.2–13.0)
Prostate weight after RARP, g, median (IQR)	30.5 (22.1–43.3)
Follow up, months, median (IQR)	28.2(14.1–51.8)
**ISUP grade at biopsy, %**	
1	44.0
2	17.5
3	16.6
4	13.2
5	8.7
**Pathological T stage, %**	
T2	43.4
T3a	44.8
T3b	11.8
**ISUP grade at RP, %**	
1	15.0
2	33.7
3	32.0
4	5.3
5	14.0

*RARP* robot-assisted radical prostatectomy, *BMI* body mass index, *PSA* prostate-specific antigen, *ISUP* International Society of Urological Pathology, *RP* radical prostatectomy.

### The predictors of PSM

#### Preoperative predictors

Two preoperative factors (Table 2), namely PSA level (*p* < 0.001) and ISUP grade at biopsy (*p* = 0.025) were significant predictors of PSM in RP specimens. Furthermore, 39% of patients with PSA > 10 ng/mL and 43.2% of those with ISUP grade > 3 had PSM after RARP. Age (*p* = 0.84), BMI (*p* = 0.158), and clinical stage determined through MRI (*p* = 0.827) exhibited no significant difference between groups displaying positive and negative margins.

**Table 2:** Comparison of predictive characteristics between positive and negative surgical margins for 419 patients having undergone robotic prostatectomy.

Variable predictors	Negative margin	Positive margin	*p* value
	N = 293	N = 126	
**Preoperative**			
*Age*			0.840
Median (range)	66 (43–85)	66 (48–84)	
*BMI*			0.158
Median (range)	25.0 (15.2–35.5)	25.5 (17.8–33.8)	
*PSA*			< 0.001*
Median (range)	8.2 (1.0–52.5)	10.6 (4.3–89.9)	
*MRI clinical-stage, n (%)*			0.018*
≤ T2	165 (66.3)	67 (65.0)	
> T2	84 (33.7)	36 (35.0)	
*ISUP grade at biopsy, n (%)*			0.025*
1	137 (74.9)	46 (25.1)	
2	52 (70.3)	22 (29.7)	
3	48 (70.6)	20 (29.4)	
4	37 (67.3)	18 (32.7)	
5	17 (47.2)	19 (52.8)	
**Intraoperative**			
*Console time (mins)*			0.625
Median (range)	230 (154–480)	233 (160–480)	
*Estimated blood loss (ml)*			0.015*
Median (range)	27 (3–800)	30 (3–1350)	
*Surgical cases, n(%)*			0.881
001–100	71 (24.2)	29 (23.0)	
101–200	72 (24.6)	28 (22.2)	
201–300	67 (22.9)	33 (26.2)	
301–420	83 (28.3)	36 (28.6)	
*Median lobe, n (%)*			0.837
Yes	37 (12.6)	15 (11.9)	
No	256 (87.4)	111 (88.1)	
*History of TURP, n (%)*			0.936
Yes	18 (6.1)	14 (11.1)	
No	275 (93.9)	112 (88.9)	
*History of abdominal surgery, n (%)*			0.408
Yes	20 (9.7)	12 (12.9)	
No	186 (90.3)	81 (87.1)	
*Nerve-sparing, n(%)*			0.047*
Yes	282 (96.6)	116 (92.1)	
No	10 (3.4)	10 (7.9)	
**Postoperative**			
*Clavien complication, n (%)*			0.248
Grade 0	241 (82.3)	110 (87.3)	
Grade ≥ 1	52(17.7)	16 (12.7)	
*ISUP grade at RP, n (%)*			< 0.001*
1	54 (18.4)	9 (7.1)	
2	104 (35.5)	37 (29.4)	
3	89 (30.4)	45 (35.7)	
4	18 (6.1)	4 (3.2)	
5	28 (9.6)	31 (24.6)	
*Pathological T stage, n (%)*			< 0.001*
T2	159 (54.4)	22 (17.6)	
T3a	107 (36.6)	80 (27.5)	
T3b	26 (9.0)	23 (18.4)	
*Prostate weight*			
Median (range)	30.9 (6.0–124.4)	29.3 (8.4–170.4)	0.141

*BMI* body mass index, *PSA* prostate-specific antigen, *MRI* magnetic resonance imaging, *ISUP* International Society of Urological Pathology, *RP* radical prostatectomy, *TURP* transurethral resection of the prostate, *p value for difference between margins status < 0.05; statistical analysis, continuous data: *t*-test, categorical data: chi-square test.

#### Intraoperative predictors

The surgical duration of RARP did not affect the PSM rate. The estimated blood loss increased slightly but significantly (30 mL vs. 27 mL; *p* = 0.015) in the PSM group over the negative surgical margin (NSM) group. The PSM rate was steady, with approximately 30% per 100 cases (*p* = 0.881) in a single surgeon’s experience. The intraoperative factors including console time, previous abdominal surgery, previous TURP, and a prominent median lobe, displayed similar differences over the two groups.

#### Postoperative predictors

The PSM rate significantly increased at a higher pT stage (*p* < 0.001) and a higher grade of ISUP (*p* < 0.001) in RP specimens. The prostate weight in prostatectomy was similar between the two groups (*p* = 0.141). Overall, the postoperative findings indicated that the more advanced the disease’s progression was, the higher the PSM rate was upon final pathological examination.

#### Prediction of PSMs’ location and number

The percentage of PSMs (Table 3) in the apex, bladder neck, and posterolateral regions was 27.7%, 13.5%, and 73.8%. Ninety-four (74.6%) RP specimens presented unifocal, while 29 (23%) presented multifocal positive margins. The PSA level, pT stage, and ISUP grade at RP were significantly associated with PSMs in the bladder neck, posterolateral, unifocal, and multifocal regions, respectively. The amount of intraoperative estimated blood loss was significantly higher in the apex of the PSM (*p* < 0.001), whereas an enlarged prostate median lobe was significantly more common in the bladder neck of PSM (9.6% vs. 3.3%; *p* = 0.047). Moreover, the NS procedure was significantly associated with PSM in the bladder neck (*p* < 0.001) and posterolateral (*p* = 0.012) regions. Regarding the number of PSMs, BMI, previous abdominal surgery, and NS procedures were predictive factors for PSMs in multifocal regions.

**Table 3:** Predictors based on the location and number of positive surgical margins.

Variable predictors	AP	BN	PL	UF	MF
	N = 35	N = 17	N = 93	N = 94	N = 29
**Continuous data**					
*Age*					
Median (range)	68 (48–76)	65 (50–70)	66 (43–85)	66 (43–85)	67 (55–78)
*BMI*					*p = 0.025
Median (range)	25.6 (20–31)	26.3 (21–33)	25.8 (18–34)	25.0 (18–34)	26.8 (22–33)
*PSA*		*p = 0.001	*p < 0.001	*p = 0.023	*p < 0.001
Median (range)	11.6 (4–78)	11 (5–90)	10.4 (4–90)	9.7 (4–87)	12.2 (5–90)
*Prostate weight*					
Median (range)	28.1 (14–90)	29.3 (16–87)	29 (8–170)	29.6 (8–170)	28.1 (15–77)
*Operative time (mins)*					
Median (range)	230 (165–480)	215 (180–345)	235 (160–480)	234 (165–440)	230 (160–480)
*Estimated blood loss (ml)*	*p < 0.001				
Median (range)	30 (5–1350)	30 (3–550)	30 (3–1350)	32 (3–750)	30 (5–1350)
**Categorical data**					
*Biopsy ISUP score, n (%)*			*p = 0.019	*p = 0.017	
1	14 (7.7)	5 (2.7)	35 (19.1)	32 (17.5)	12 (6.6)
2	10 (13.5)	3 (4.1)	16 (21.6)	13 (17.6)	8 (10.8)
3	4 (5.9)	4 (5.9)	13 (19.1)	18 (26.5)	2 (2.9)
4	4 (7.3)	1 (1.8)	12 (21.8)	17 (30.9)	1 (1.8)
5	3 (8.3)	4 (11.1)	16 (44.4)	14 (38.9)	5 (13.9)
*RP ISUP score, n (%)*		*p = 0.005	*p = 0.001	*p = 0.005	*p = 0.032
1	4 (6.3)	0 (0)	6 (9.5)	10 (15.9)	0 (0)
2	12 (8.5)	3 (2.1)	27 (19.1)	23 (16.5)	11 (7.8)
3	14 (10.4)	7 (5.2)	32 (23.9)	34 (25.4)	10 (7.5)
4	0 (0)	0 (0)	4 (18.2)	4 (18.2)	0 (0)
5	5 (8.5)	7 (11.9)	24 (40.7)	23 (39.0)	8 (13.6)
*MRI clinical Stage, n (%)*					
≤ T2	20 (8.6)	6 (2.6)	48 (20.7)	50 (21.6)	15 (6.5)
> T2	7 (5.8)	8 (6.7)	27 (22.5))	30 (25)	6 (5)
*Pathological T stage, n (%)*		*p < 0.001	*p < 0.001	*p < 0.001	*p < 0.001
T2	17 (9.4)	0 (0)	10 (5.5)	20 (11.0)	3 (1.7)
T3	18 (7.6)	16 (6.8)	82 (34.7)	74 (31.4)	25 (10.6)
*Clavien complication, n (%)*					
Grade 0	31 (8.8)	15 (4.3)	81 (23.1)	81 (23.1)	25 (7.1)
Grade ≥ 1	4 (5.9)	2 (2.9)	12 (17.6)	13 (19.1)	4 (5.9)
*Median lobe, n (%)*		*p = 0.047			
Yes	3 (5.8)	5 (9.6)	9 (17.3)	13 (25.0)	2 (3.8)
No	32 (8.7)	12 (3.3)	84 (22.9)	81 (22.1)	27 (7.4)
*History of TURP, n (%)*					
Yes	0 (0)	1 (3.8)	7 (26.9)	4 (15.4)	3 (11.5)
No	35 (8.9)	16 (4.1)	86 (21.9)	90 (22.9)	26 (6.6)
*History of abdominal surgery, n (%)*					*p = 0.018
Yes	3 (9.4)	2 (4.1)	11 (34.4)	6 (18.8)	6 (18.8)
No	19 (7.1)	11 (6.2)	62 (23.2)	59 (22.1)	18 (6.7)
*Nerve-sparing, n (%)*		*p < 0.001	*p = 0.012		*p = 0.018
Yes	34 (8.5)	13 (3.3)	84 (21.1)	88 (22.1)	25 (6.3)
No	1 (5)	4 (20)	9 (45)	6 (30)	4 (20)
*Surgical cases, n (%)*					
001–100	11 (11)	4 (4)	18 (18)	25 (25)	5 (5)
101–200	9 (9)	2 (2)	19 (19)	20 (20)	4 (4)
201–300	6 (6)	7 (7)	27 (27)	24 (24)	9 (9)
301–420	9 (7.6)	4 (3.4)	29 (24.4)	25 (21)	11 (9.2)

*PSA* prostate-specific antigen, *BMI* body mass index, *ISUP* International Society of Urological Pathology, *RP* radical prostatectomy, *TURP* transurethral resection of the prostate, *AP* apex, *BN* bladder neck, *PL* posterolateral, *UF* unifocal, *MF* multifocal, *p value for difference between margins status < 0.05; statistical analysis, continuous data: *t*-test, categorical data: chi-square test.

### The multivariable analysis of PSM’s predictors

In multivariable analysis (Table 4), the pT3 stage, PSA > 10 ng/mL, and estimated blood loss > 200 mL were significant predictors for PSM rates (*p* < 0.001, odds ratio [OR] 6.901; *p* = 0.047, OR 1.712; and *p* = 0.001, OR 4.010, respectively). Conversely, a higher clinical T (cT) stage was significantly associated with a lower PSM rate (*p* = 0.040, OR 0.058), which was in contrast with the effect of the pT stage on the margin status. The ISUP grade at biopsy (*p* = 0.130) or RP specimens (*p* = 0.787) displayed a similar ratio between PSM and NSM groups. On comparing the NS and non-NS methods, no significant changes in the PSM rate were observed (*p* = 0.742).

**Table 4:** Analysis of the primary parameters to predict positive surgical margins (PSM) after robotic prostatectomy by univariable analysis, and multivariable logistic regression analysis.

PSM predictive parameters	Univariable	Multivariable	*p* value
	*p* value	Odds ratio (95% CI)	
PSA > 10 ng/ml	0.001	1.712 (1.008–2.907)	0.047*
EBL > 200 ml	0.011	4.010 (1.496–10.752)	0.006*
cT3 stage	0.018	0.548 (0.308–0.974)	0.040*
pT3 stage	< 0.001	6.901 (3.624–13.142)	< 0.001*
ISUP at biopsy > 3	0.012	1.656 (0.862–3.180)	0.130
ISUP at RP > 3	0.004	0.908 (0.449–1.833)	0.787
NS procedure	0.047	0.825 (0.261–2.602)	0.742

*PSA* prostate-specific antigen, *EBL* estimated blood loss, *cT* clinical T stage by MRI, *pT* pathological stage by RP specimens, *ISUP* International Society of Urological Pathology, *RP* radical prostatectomy, *NS* nerve-sparing, *p value for difference between margins status in multivariable analysis < 0.05.

### The predictors of BCR

#### Operative predictors

In total, 395 (94.3%) patients were available for follow-up evaluation, and 97 (24.6%) patients developed recurrent PSA. The overall 5-year BCR-free survival rate was 66.7%. In univariate analysis (Table 5), the initial predictors of PSA, BMI, cT stage, pT stage, PSM, NS procedures, ISUP grade at biopsy, and RP were significantly correlated with the 5-year BCR-free survival rate.

**Table 5:** Analysis of the primary predictors of 5-year biochemical recurrence-free survival by the log-rank test in univariable analysis and multivariable Cox regression models.

BCR predictive parameters	Univariable	Multivariable	*p* value
	*p* value	Hazard ratio (95% CI)	
PSA > 10 ng/ml	< 0.001	1.801 (1.123–2.888)	0.015*
pT3 stage	< 0.001	2.264 (1.199–4.275)	0.012*
PSM	< 0.001	1.725 (1.065–2.792)	0.027*
ISUP at RP > 3	< 0.001	1.964 (1.111–3.472)	0.020*
ISUP at biopsy > 3	< 0.001	0.809 (0.463–1.413)	0.809
cT3 stage	< 0.001	1.548 (0.944–2.536)	0.083
NS procedure	0.001	0.518 (0.240–1.118)	0.094
BMI > 25 kg/m^2^	0.012	1.176 (0.744–1.859)	0.487

*BCR* biochemical recurrence, *PSA* prostate-specific antigen, *pT* pathological T, *PSM* positive surgical margin, *ISUP* International Society of Urological Pathology, *RP* radical prostatectomy, *cT* clinical T, *NS* nerve-sparing, *BMI* body mass index. *p value for difference between biochemical recurrence in multivariable analysis < 0.05.

#### PSMs’ impact on BCR

**Figure 1:**
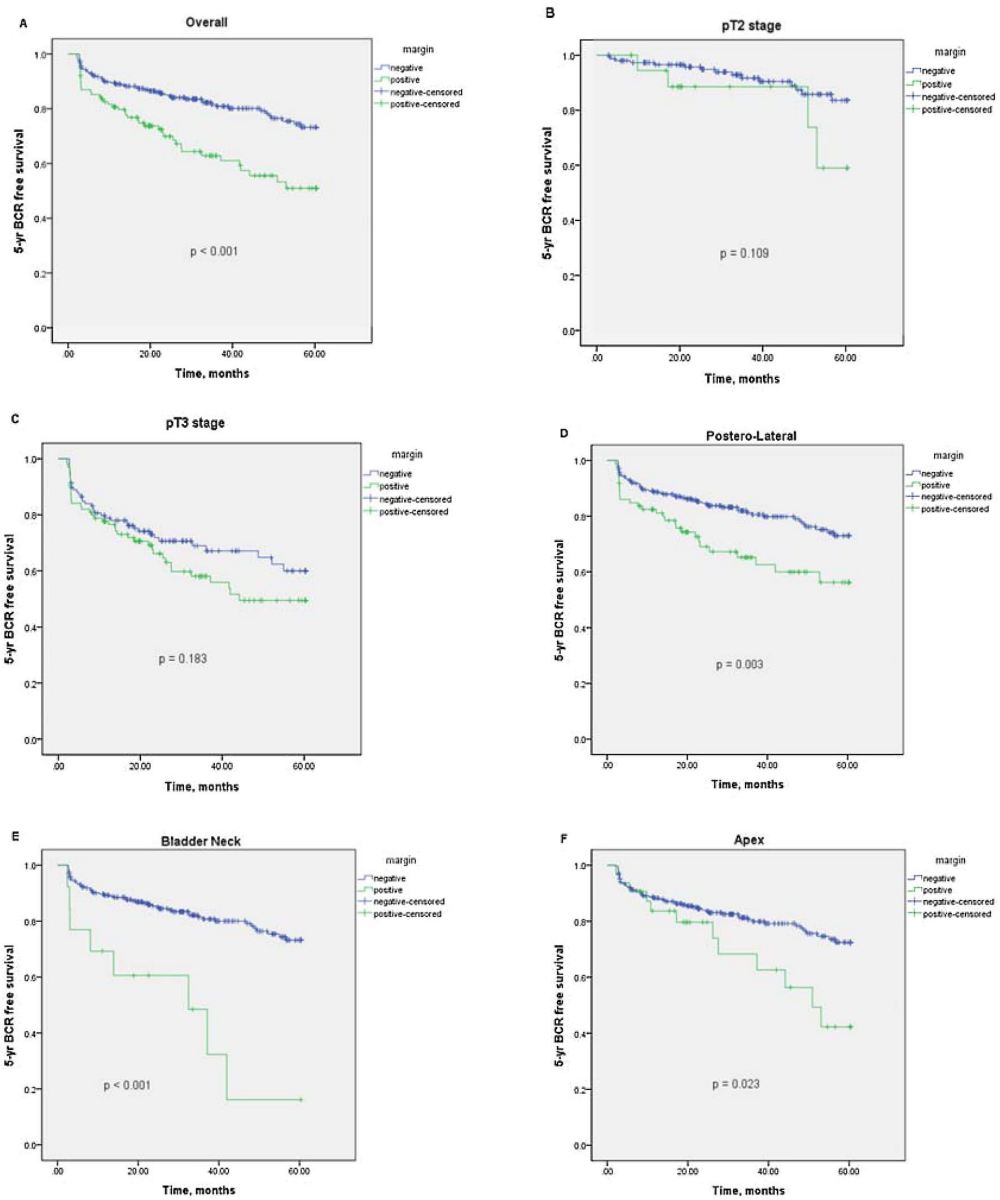
Kaplan–Meier curve for 5-year biochemical recurrence (BCR)-free survival stratified by margin status. (**A**) stratified by positive and negative surgical margins (*p* < 0.001); (**B**) and (**C**) stratified by pT2 and pT3 stage (*p* = 0.109 and *p* = 0.183); **(D**)–(**F**) stratified by positive surgical margins in posterolateral, bladder neck and apex (*p* = 0.003, *p* < 0.001 and *p* = 0.023).

**Figure 2:**
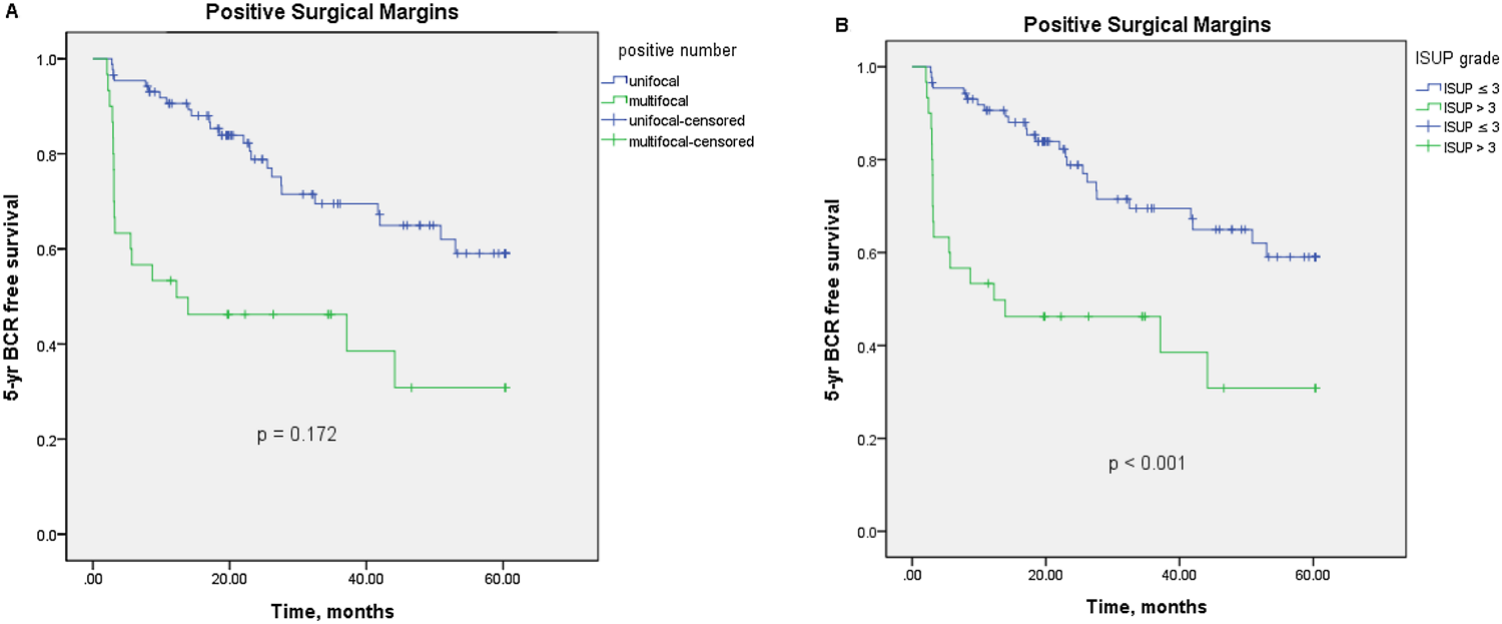
Kaplan–Meier curve for 5-year biochemical recurrence (BCR)-free survival in cases with positive surgical margins (**A**) stratified by unifocal and multifocal positive al margins (*p* = 0.172); (**B**) stratified by ISUP grade > 3 and ISUP grade ≤ 3 (*p* < 0.001).

Among patients with a PSM, the 1-, 3-, and 5-year BCR-free survival rates were 79.7%, 61.1%, and 41.9%; by comparison, those with an NSM were 88.9%, 80.9%, and 71.4%, respectively. Figure 1 indicated that PSM's presence significantly decreased the 5-year BCR-free survival rate overall (*p* < 0.001). However, the presence of PSM did not decrease the 5-year BCR-free survival rate when stratified by pT2 and pT3 stages**.** Concerning the location of PSMs, positive margins in apical, bladder neck and posterolateral regions significantly reduced 5-year BCR-free survival (*p* = 0.003, *p* < 0.001 and *p* = 0.023, respectively). Figure 2 further evaluated the covariate factors among PSMs’ group and revealed the 5-year BCR-free survival curve was similar in the multifocal and unifocal groups (*p* = 0.172). However, the ISUP grade > 3 subgroup had a lower 5-year BCR-free survival rate than the ISUP grade ≤ 3 subgroup (*p* < 0.001).

### The multivariable analysis of BCR’s predictors

In multivariable analysis (Table 5), only four independent predictors, namely PSA > 10 ng/mL (*p* = 0.015, hazard ratio [HR] 1.801), pT3 stage (*p* = 0.012, HR 2.264), ISUP grade > 3 at RP (*p* = 0.020, HR 1.964), and PSM (*p* = 0.027, HR 1.725), were positively correlated with PSA recurrence. When pooling four independent predictors during multivariable analysis, the PSM parameter became less significant in predicting the 5-year BCR-free survival rate (Table 6). In pT2 stage disease, no significant predictors of BCR-free survival were identified. For pT3 stage disease, except PSM (*p* = 0.323), both PSA > 10 ng/mL (*p* = 0.025) and ISUP grade at RP > 3 (*p* = 0.002) were significantly associated with BCR-free survival. Among patients with an NSM, the predictors of PSA > 10 ng/mL (*p* = 0.006), ISUP grade at RP > 3 (*p* = 0.001), and pT3 stage (*p* = 0.003) significantly influenced BCR-free survival. However, for PSM’s patients, an ISUP grade > 3 detected in RP specimens was the only significant predictor of BCR-free survival (*p* = 0.002, HR 2.689).

**Table 6:** Analysis of the primary parameters classified based on pathological stage and margin status to predict the 5-year biochemical recurrence-free survival through multivariable Cox regression analysis.

Predictive parameters	Hazard Ratio (95% CI)	*p* value
**Overall (n = 417)**		
ISUP at RP > 3	2.727 (1.776–4.185)	< 0.001*
pT3 stage	2.510 (1.441–4.370)	0.001*
PSA > 10 ng/ml	1.660 (1.092–2.523)	0.018*
PSM	1.365 (0.895–2.081)	0.149
**pT2 stage (n = 181)**		
ISUP at RP > 3	1.384 (0.180–10.618)	0.775
PSA > 10 ng/ml	2.393 (0.784–7.305)	0.126
PSM	1.504 (0.566–3.994)	0.413
**pT3 stage (n = 236)**		
ISUP at RP > 3	2.851 (1.816–4.476)	0.002*
PSA > 10 ng/ml	1.710 (1.069–2.737)	0.025*
PSM	1.251 (0.802–1.952)	0.323
**NSM (n = 293)**		
ISUP at RP > 3	2.744 (1.523–4.944)	0.001*
pT3 stage	2.615 (1.379–4.956)	0.003*
PSA > 10 ng/ml	2.218 (1.379–4.956)	0.006*
**PSM (n = 126)**		
ISUP at RP > 3	2.689 (1.428–5.067)	0.002*
pT3 stage	1.655 (0.564–4.856)	0.359
PSA > 10 ng/ml	1.158 (0.630–2.130)	0.636

*pT* pathological T, *PSA* prostate-specific antigen, *ISUP* International Society of Urological Pathology, *RP* radical prostatectomy, *NSM* negative surgical margin, *PSM* positive surgical margin, *p value for difference between biochemical recurrence in multivariable analysis < 0.05.

## Discussion

RARP offers potential benefits such as a low PSM rate compared with open RP owing to better visibility and less blood loss^19,20^. Previous studies have discussed the prediction of PSM after RARPs using single-surgeon case study^21,22^. The benefit of single-surgeon case series is taking a similar surgical approach to decrease the variation and bias as compared with other studies that retrieved the RARPs performed by plenty of urologists with different methods from various centers. In our series, the overall PSM rate was 30.1%, which is a higher rate than that reported in high-volume RARP studies, in which the range typically was 10.8–22%^22^. This may be attributable to a much higher percentage (56.6%) of patients in the pT3 stage in our Asian study than in other Western studies, where the percentage of pT3 stage patients ranged from 9.3 to 37.5%^22^.

We determined the major preoperative predictor for the PSM rate to be PSA > 10 ng/mL. The predictors of the ISUP score upon biopsy and the cT stage defined through MRI did not exhibit a positive correlation in multivariable analysis. Liss et al. assessed 216 cases of RARPs and concluded, similar to us, that the preoperative predictive factor of PSM was PSA level instead of cT stage and ISUP score^23^. The author explained this phenomenon because a portion of patients was transferred from other hospitals; thus, the number of biopsies and MRI outcomes were not standardized, potentially yielding different results. Another reason we proposed may be the high pathological ISUP (43%) and T stage (30.2%) upgrading rate from biopsy to prostatectomy in our database**.** Our previous study reports the pathological upgrading rate was about 35.5% after prostatectomy for the patients who underwent more than 10 cores of biopsy. The disconcordant rate was higher for the patients who received nonextended than extended biopsies^24^. Gleason had described in 1992 that “the limited amount of tissue obtained in needle biopsy which may let the pathologist hesitate to interpret the higher grade cancer if the amount of tumor is very small.” These factors may limit the precise prediction of the postoperative PSM rate through the use of the preoperative cT stage and ISUP grade.

Estimated blood loss > 200 mL is a significant intraoperative predictor of the PSM rate, and we speculated that intraoperative bleeding would hinder clear visualization from identifying prostate margins and prolonging the surgical time. In our study, further analysis found the console time was longer among patients with an estimated blood loss of > 200 mL than in those with an estimated blood loss of ≤ 200 mL (mean: 303 min vs. 235 min, respectively). Although we didn’t find a strong correlation between higher pT stage and higher estimated blood loss, the current data reported the group of prostate weight > 50 g had a higher amount of blood loss than that of prostate weight ≤ 50 g (mean 117 mL vs. 53 mL, *p* < 0.001) during prostatectomy. Kim et al. determined that higher blood loss was associated with larger prostate size in a series of 1168 RARPs. Nevertheless, the final PSM rate did not significantly differ among divided-size subgroups^8^. Tamhankar et al. reviewed 1406 RARPs to determine the steepness of the surgical learning curve reflected by the extent of blood loss and reported a 70% reduction in blood loss from the start to the end of the training period. However, the surgical time and the number of cases were not associated with the risk of PSM^25^.

Individualized surgical experiences may influence RARP performance. Several studies have reported that the PSM rate is inversely proportional to the number of surgical cases^26–29^. In our study, a single surgeon with extensive prior experience in laparoscopic surgery performed the surgeries; consequently, the PSM rate was almost the same at approximately 30% per 100 cases. This finding suggests that the surgeon’s previous experience in minimally invasive surgery can minimize the risk of PSMs when using a robotic surgical approach. White et al. reported a single-urologist case series and revealed that extensive previous experience in ORP might potentially prevent an increase in the PSM rate during the initial learning curve in RARP^30^.

The postoperative pT3 stage was significantly correlated with a high PSM rate according to previous reports^22,23,31–34^. Ficarra et al. studied 322 RARPs and reported that the pathological findings of extraprostatic extension (EPE) were the only relevant PSM predictor^35^. Previous studies have reported that T upstaging rates varied widely from 4.5 to 68% of cases^36,37^. In current study, T upstaging occurred in one-third of cases, suggesting that preoperative understaging data may result in underresection of prostate tumors. In particular, the ISUP grade at RP is not considered an independent predictor, implying that total resection of high-grade tumors can be accomplished without leaving positive margins.

Kang et al. reported the distribution of surgical margins in high-risk PCa, with positive rates of 16.2% in the apex, 14.7% in the bladder neck, 38.2% in posterolateral regions, and 26.5% in multifocal regions^31^. For our study, the majority of PSM is 73.8% in the posterolateral, 27.7% in apical, and 13.5% in bladder neck regions. Previous studies have reported that the high PSM rate occurs in the posterolateral area, especially for high-risk pT3 diseases^31,38^. Eastham et al. reported large amounts of neurovascular tissue over the posterolateral region, potentially enhancing tumor cell migration and promoting local invasion^39^; furthermore, the BCR rate was higher among individuals with posterolateral PSM than in those with NSM (HR: 2.80). The NS procedure is associated with the PSM in the posterolateral region^23,40^, potentially explaining the high technical skill needed for successful dissection of the correct planes of fascia. However, the current results demonstrated that the NS group had a lower risk of PSM in the posterolateral region than the non-NS group did (3% vs. 20%). This is probably because of the higher percentage of patients in the pT3 stage than in the pT2 stage among the non-NS group (79% vs. 21%), which may be more influential than NS techniques.

Previous studies have been reported the apex is the most frequent region of PSM in RP specimens^32,41^. This is attributable to the unclear prostate capsular margins, which are difficult to identify in pT2 and pT3 stages^38^. More other studies have reported that apical PSM is correlated with the surgeon’s approach and skills rather than the tumor stage^22,27,41^. The well-experienced surgeon in this study might explain the relatively low rate of PSM in the apex. The estimated blood loss was significantly higher among patients with apical PSM, which implies potential bleeding from the dorsal vein complex upon dissection of the apical prostate^42^. Coelho et al. reported that high BMI was a predictor for apical PSM in a cohort study involving 876 RARPs^32^, and our study determined that high BMI was significantly associated with higher odds of PSMs at multifocal regions (*p* = 0.025) but not apical regions.

Koizumi et al. reported that employing the RARP approach has a higher likelihood of leading to PSM at the bladder neck than either ORP or LARP do^30^. This is possible because of the excessive preservation of bladder neck tissue to secure postoperative urinary continence^15^. Bellangino et al. reported the PSM rate in the bladder neck ranged 0 to 16%, which was affected by the extent of bladder neck preservation^43^. The PSM rate in the bladder neck accounted for only 4.1% in our study which might attribute to the surgeon highly selected the bladder neck preservation procedure only in cases without cancer involvement over the prostatic base. Furthermore, the presence of a prominent median lobe during surgery might increase the risk of PSM over the bladder neck (*p* = 0.047), indicating the challenging task of identifying surgical margins between the protruding prostatic lobe and bladder neck.

Several studies have reported patients who underwent prostatectomy with a 5-year BCR-free survival rate ranging from 74 to 87% and a median PSA recurrence time of 2.6 years^44–46^. In this study, the 5-year BCR-free survival rate was 66.7%, which was lower than that reported in other studies; this is probably owing to the higher ratio of aggressive pT3 disease at the outset of the accumulation of RARP cases. Evidence supports the characterization of PSM as a strong predictor of disease progression^47–49^. Recently, Zhang et al. performed a systematic review and meta-analysis, wherein they included 38,000 patients and determined PSM to be an independent factor with higher BCR in multivariable analysis (*p* < 0.001, pooled HR 1.35)^6^. Ploussard et al. analyzed a prospective study including 1943 RPs with a mean follow-up of 68 months and suggested that PSM was a significant predictor of BCR, the need for salvage therapy, and even cancer-related death. However, PSM was significantly correlated with BCR at stage pT2 and pT3a disease but not pT3b stage disease^49^. PSM's effect on BCR in stage pT3b disease was reportedly weak owing to a markedly higher risk of micrometastatic lesions, which are more influential than PSM is. Our data indicate that the hazard ratio for PSM with BCR is 1.725 (*p* = 0.027). However, no strong evidence suggests the PSM has a strong impact on BCR in separate pT2 and pT3 comparisons. The main predicting factor is the pT stage rather than the effect of PSM on biochemical recurrence.

In particular, among men who underwent RARP with postoperative PSM, an ISUP grade > 3 at RP (*p* = 0.002, HR 2.689) was the sole predictor of BCR-free survival regardless of pT stage and PSA concentration. Karakiewicz et al. reported similar results among 5831 RPs, indicating that the PSM group had a 3.7-fold higher risk of progression, particularly in the group with tumors at an ISUP grade > 2 in PSM^48^. Furthermore, Kang et al. reported that pathological ISUP grade > 3 was a predictor for BCR (*p* = 0.047, HR 4.180). These results suggest that disease progression depends on the PCa tumor grade in surgical margins^50^. Moreover, Stephensen et al. reported the effect of the number and extent of PSM on BCR, indicating that a mildly increased risk of BCR is significantly correlated with multifocal and extensive PSM^51^. However, we did not analyze the effect of PSM's extent; the current evidence is inadequate to differentiate the effect of unifocal and multifocal PSM on BCR-free survival. The residual low-grade tumors as compared to high-grade tumors on PSM may not increase the BCR rate. Our study’s surgeon used electrocautery methods when dissecting the prostate fascia, thus indirectly decreasing the residual tumors on margins and reducing PSA recurrence risk.

A collaborative study shows the pathological grade and stage of diagnosed PCa in Asia countries is more aggressive than that in Western countries^52^. However, much-developed Asian areas such as Korea, Singapore, and Hong-Kong, had similar characteristics of PCa in Western countries. Overall, detection rate, environmental and genetic factors may contribute to the reason^52,53^. For the prediction of PSM, previous Western studies mostly reported preoperative PSA, clinical stage, and ISUP grade are the main correlative factors^23,54,55^. Additionally, these three preoperative factors are predictors in the Partin table which is a nomogram developed by John Hopkins Hospital to predict EPE (pT3 stage)^56^ in Western countries. However, in our data, only preoperative PSA level was significantly associated with PSM and EPE in multivariable analysis. The discrepancy of clinical stage and ISUP upgrading between biopsy and RP specimens may reduce the utility of these two preoperative parameters for predicting PSM. Nevertheless, postoperative pT3 pathology can not be served as a useful predictor before surgery but indeed a high correlation between the pT3 stage and PSM is expected. Tian et al. developed a nomogram to predict PSM in the Chinese population and disclosed a higher PSM rate and pT3 stage in their Asian study group than that in Western countries group. They reported the clinical stage as a strong predictor for PSM and emphasized the key of clinical-stage interpretation obtained by both MRI and digital-rectal examination which may be insufficient in our current study^57^.

For the prediction of BCR, previous three Western^58–60^, as well as one Asian^61^ studies, discovered PSA, EPE, seminal vesicle invasion (SVI), ISUP grade, and margins status are the chief predictors in nomograms for predicting early BCR after prostatectomy. The results of predictive factors are similar in our current study. However, the difference is that we did not divide the pT3 stage into EPE or SVI, and calculated their individualized risks, separately. Therefore, the high percentage of PCa with pT3 stage in our finding may surpass the impact of PSM on BCR. Especially when we analyze the data and restrict it to only pT3 cases, the PSM is not considered a strong factor for predicting BCR. The pathological T3 stage is the most powerful predictor for PSA recurrence.

### Limitations

First, since we retrospectively obtained clinical data, there may have been an inherent selection bias in the analysis. Second, the lack of standardized MRI and pathological interpretations from other hospitals may affect the results in prostatectomy. Third, the results were obtained from a single experienced surgeon; thus, the technical details regarding NS methods and the determination of prominent prostate median lobes may have led to subjective bias. Fourth, we determined prostate size by using the data from RP specimens instead of presurgical imaging, which may have restricted the clinical application of this method for preoperative assessment. Finally, some cases were lost to follow-up early during the study, and some clinically significant diseases recurred after five years. The current follow-up duration was limited for the prediction of the final endpoint of BCR.

## Conclusion

This is an Asian population-based study to precisely overview the predictors of PSM and BCR on RARP. Our results indicated that PSA level, pT stage, and estimated blood loss were the significant predictors of PSM. Regarding 5-year BCR-free survival, PSA, pT stage, ISUP grade, and PSM were identified as clinically significant predictors in multivariable analysis. The NS procedure was associated with the posterolateral PSMs while intraoperative bleeding was correlated with apical PSMs. However, our Asian population-based study showed a high ratio of PCa having pT3 stage which might surpass the impact of PSM on BCR. PSM was less significant than PSA and ISUP grade for predicting BCR in pT3 disease. Nevertheless, in cases of PSM observed after prostatectomy, unifocal and multifocal PSM had a similar ratio to the BCR rate but ISUP grade > 3 was the sole predictor of PSA recurrence.
